# Gallstone Ileus: Uncommon Presentation Followed by Less Common Spontaneous Resolution

**DOI:** 10.7759/cureus.12138

**Published:** 2020-12-18

**Authors:** Nicholas Hobbs, Mohammed Barghash, Paul A Peters, Moustafa Mansour

**Affiliations:** 1 General Surgery, North Manchester General Hospital, Manchester, GBR

**Keywords:** gallstone, ileus, gallstone ileus, spontaneous resolution, bowel obstruction, resolution

## Abstract

Gallstone ileus is a rare but significant cause of bowel obstruction. An 82-year-old female was admitted to the hospital with abdominal pain and was initially treated for a possible urinary tract infection. Following a surgical review and based on history, clinical examination as well as radiological findings, a diagnosis of gallstone ileus was made. The patient was prepared for surgery; however, whilst awaiting theatre, she spontaneously passed the obstructing gallstone with full resolution of bowel obstruction symptoms. The usual treatment for gallstone ileus is surgical management with an enterolithotomy; nevertheless, this case highlights the importance of close monitoring and adapting a management plan to fit an evolving clinical scenario.

## Introduction

Gallstone ileus is an uncommon cause of bowel obstruction. It is estimated to account for 1% to 4% of all mechanical bowel obstruction cases. However, this percentage tends to increase up to approximately 25% in patients above the age of 65 [1]. It is more prevalent in women than men (a ratio between 1:3 and 1:7) and those of Caucasian ancestry (accounting for 67% of gallstone ileus patients) [2].

The mortality of gallstone ileus ranges from 7% to 30% (average 18%). This high mortality is attributed to other factors such as being elderly or frail, having multiple co-morbidities (particularly cardiovascular, respiratory and endocrine including diabetes and obesity) and a late presentation from the onset of symptoms (on average between 4 and 8 days). Delayed diagnosis is also a feature attributing to a worse outcome [2].

Gallstone ileus is an infrequent complication of cholelithiasis and is characterised by the impaction of one or more gallstones within the lumen of the gastrointestinal (GI) tract. The occurrence of this impaction is determined by the size of the gallstone and is dictated by the site of fistula formation to the bowel. The majority of gallstones smaller than 2 to 2.5 cm may pass spontaneously through a normal GI tract uneventfully [3]. However, gallstones leading to obstruction can range anywhere from 2 to 10 cm with a mean value of 4.3 cm [4]. Stones larger than 5 cm are more likely to become impacted with the largest gallstone causing intestinal obstruction measuring 17.7 cm in its diameter [5]. This was surgically removed from the transverse colon [6].

Gallstone ileus is usually preceded by an initial bout of cholecystitis. Inflammation to the wall of the gallbladder and surrounding structures precipitates the formation of adhesions [4]. Gallstones that are large enough to cause bowel obstruction would not be able to gain access to the GI tract through a normal anatomical route. Anatomical routes would require stones passing down the cystic duct, common bile duct and through the ampulla of Vater - an opening too small to allow this passage. Instead, the pressure effect of the culprit gallstone(s) may gradually erode through the gallbladder wall. This can lead to the development of a fistula between the gallbladder and surrounding adhered parts of the GI tract. More often, this is the duodenum due to its close proximity. Once within the duodenum, the gallstone may pass spontaneously through the rectum, or it may become impacted and cause bowel obstruction. Less commonly, if the gallstone is in the stomach, then proximal migration can occur and the gallstone may be vomited [6].

Here we present a case of gallstone ileus caused by a 3-cm gallstone in a patient with an initial diagnosis of kidney injury that spontaneously resolved whilst awaiting surgical intervention.

## Case presentation

An 82-year-old Caucasian lady presented to the Emergency Department of a busy District General Hospital experiencing lower abdominal symptoms. These symptoms included two days of intermittent, colicky abdominal pain that was more intense on the left side and was associated with reduced oral intake. She had noticed episodes of dysuria, a decreased urine output and was intermittently vomiting. She last opened her bowels two days prior to presentation. Her inflammatory markers were mildly raised (white blood cells 13.8 x 10^9^/L, C-reactive protein 19 mg/L) and she showed signs of an acute kidney injury on a background of chronic kidney disease (acute-on-chronic kidney disease [AoCKD]) based on an estimated glomerular filtration rate (eGFR) of 25 mL/min/1.73 m^2^ (her baseline was 45-50).

Her observations were within normal limits with a temperature of 37.1°C, blood pressure of 124/73, heart rate of 73, respiratory rate of 17 per minute and oxygen saturation of 95% in room air, and her Glasgow Coma Scale (GCS) score was 15. On examination, her abdomen was soft and mildly diffusely tender. The tenderness was worse in the left iliac fossa. A bladder scan revealed a volume of 27 mL. Her past medical history included atrial fibrillation, chronic kidney disease, hypertension and previous hip replacement.

There was no past surgical history of abdominal procedures/surgeries. She regularly took apixaban, digoxin, ramipril, bendroflumethiazide and bisoprolol. She was independent, living alone and managing all daily activities well without assistance.

The patient was started on IV fluids and referred to the medical team. She was provisionally diagnosed with a urinary tract infection and AoCKD secondary to dehydration. Antibiotic treatment was initiated. An ultrasound scan (USS) of her kidneys, ureters and bladder (KUB) was performed; her nephrotoxic medications were withheld and she was catheterised with hourly volumes recorded.

**Figure 1:**
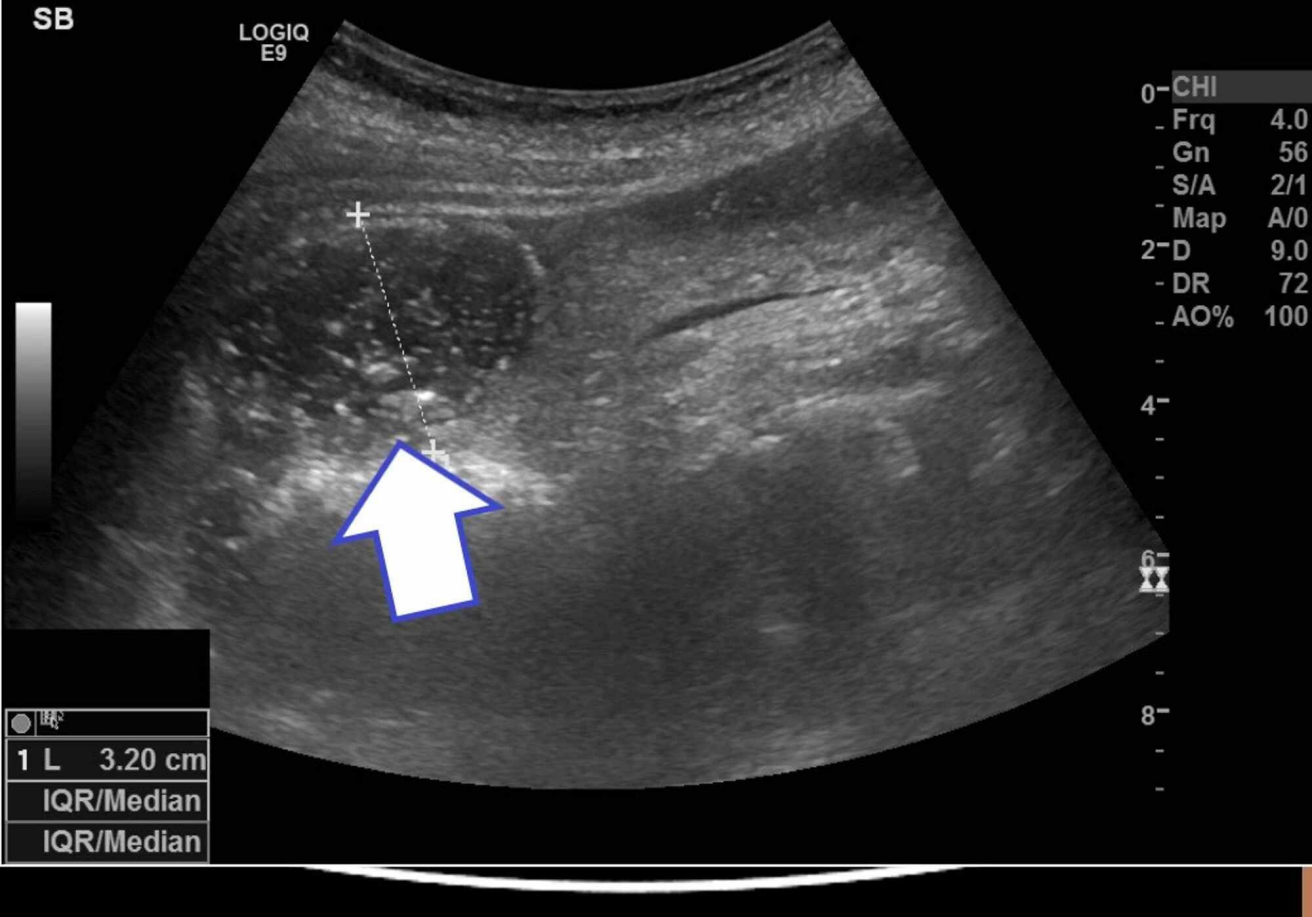
An ultrasound scan showing small bowel distension measuring 3.2 cm (white arrow indicates the distended small bowel)

The USS KUB revealed appearances of an unremarkable urinary tract. However, it did demonstrate markedly dilated loops of small bowel (Figure 1) with a seemingly normal colon. The gallbladder was thick walled and contained multiple gallstones. There was a small amount of intraluminal gas present. A segment of dilated small bowel was in close proximity to the gallbladder, hence raising the possibility of a cholecysto-duodenal fistula.

**Figure 2:**
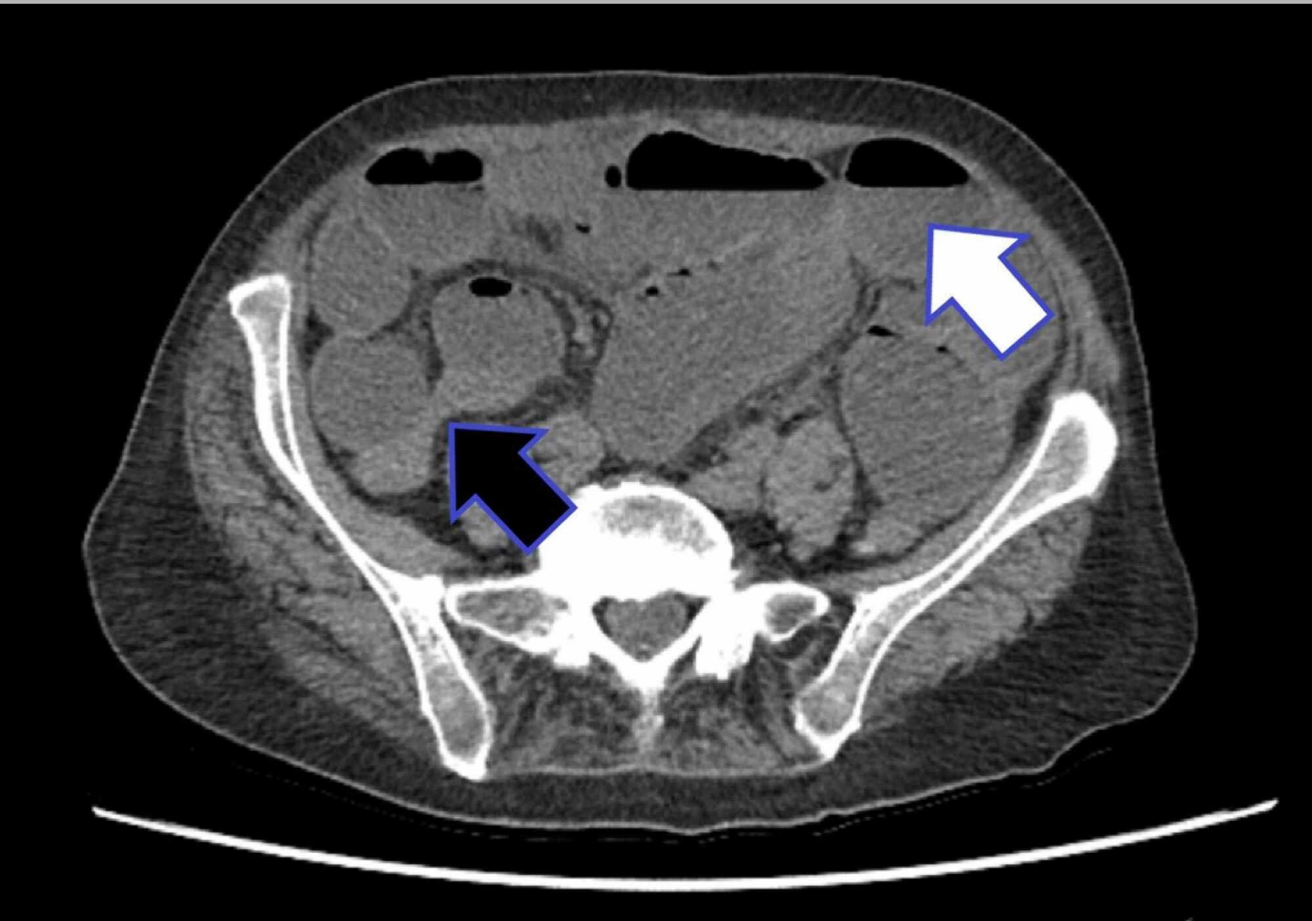
An axial cut CT scan showing small bowel dilation consistent with small bowel obstruction (white arrow indicates distended, fluid- and gas-filled small bowel; black arrow indicates the transition point at the ileocaecal valve)

**Figure 3:**
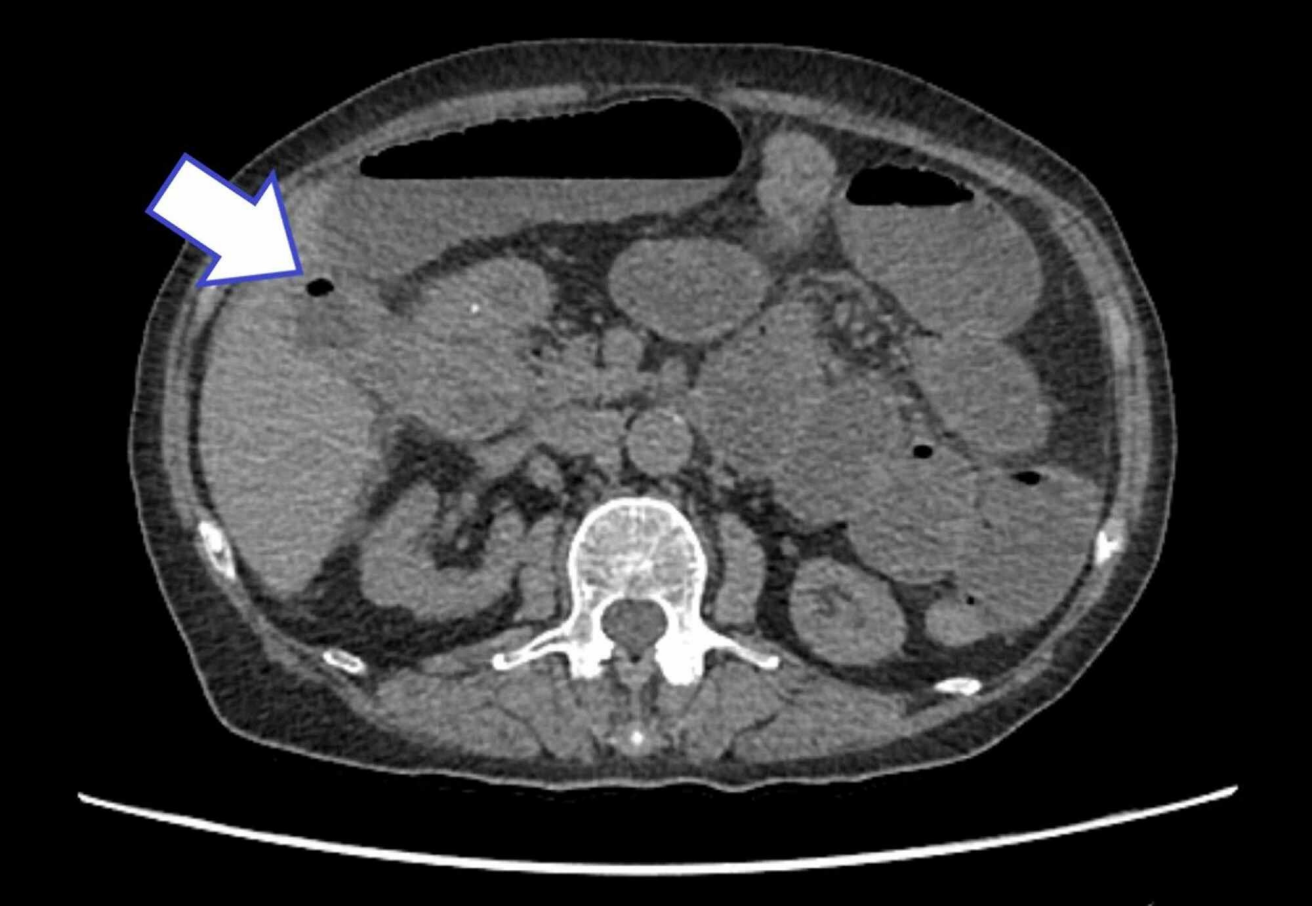
An axial cut CT scan showing air in the gallbladder fundus (white arrow indicates air within the gallbladder)

In view of her low eGFR and the results of the USS, a non-contrast computed tomography (CT) scan was performed. This confirmed distal small bowel obstruction with a transition point at, or very close to, the ileocaecal valve (Figure 2). It also revealed gas at the fundus of the gallbladder (Figure 3).

A nasogastric (NG) tube was inserted with 250 mL of gastric content draining immediately. The surgical team subsequently reviewed the patient. Given the combination of the patient’s virgin abdomen, history, clinical and radiological findings, a working diagnosis of gallstone ileus was made.

As the patient was well, had normal observations (using the National Early Warning Score system) and her abdomen was now soft and non-tender with no signs of peritonism, surgery was planned for the following morning in order to reduce the risks of operating out of hours. Haematology advice was sought with regard to the reversal of her apixaban. She was prepped, booked and consented for theatre as a priority patient for the next morning.

**Figure 4:**
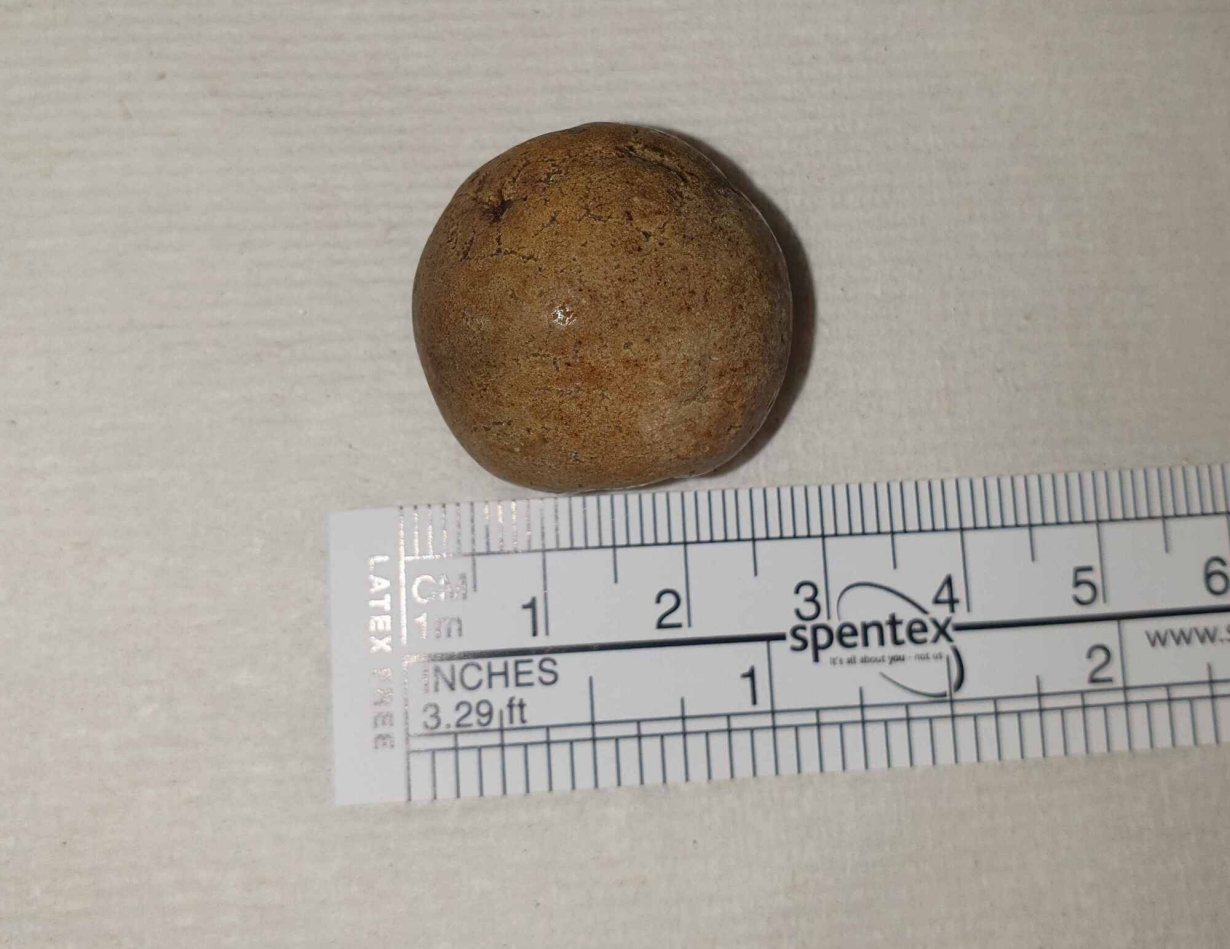
A photograph showing the obstructive gallstone following expulsion

**Figure 5:**
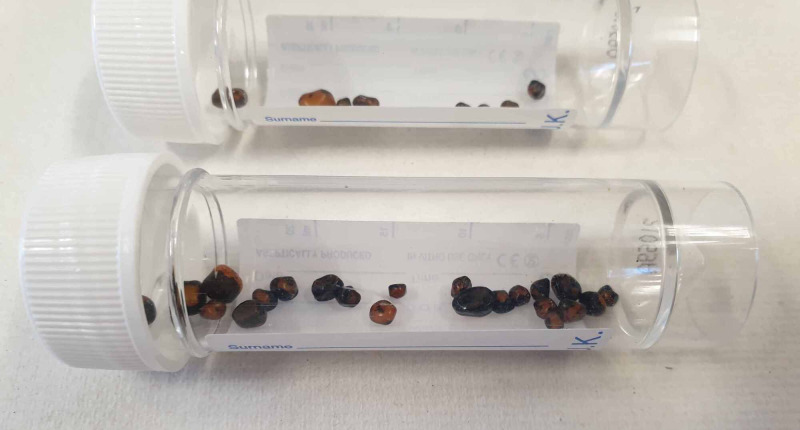
A photograph showing multiple small gallstones found in patient’s faeces

In the early morning, the patient was reviewed. She was found to have opened her bowels seven or eight times (type 7 stool) overnight and had symptomatically improved. Her pain had eased and she was no longer feeling nauseated. Staff were able to retrieve the likely offending gallstone (Figure 4) that was passed per rectum, which measured 3 cm. Following this, multiple innumerable smaller gallstones were also retrieved (Figure 5).

The patient tolerated free fluids throughout the day following the passage of calculi. On questioning, the patient did admit that she had a long history of occasional right upper quadrant pain, particularly after eating. She continued to improve clinically; the NG tube was removed the following day and she immediately tolerated a light diet consisting of soft foods. Her eGFR returned to her baseline, her oral intake was subsequently normal and she was discharged two days following the passage of the initial causative stone with appropriate safety netting instructions. An outpatient follow-up review was arranged for six weeks post-discharge.

## Discussion

Gallstone ileus is a rare disease that predominantly affects elderly, female patients [2]. This gender predominance is likely related to the fact that women are more prone to develop gallstones [7]. Due to the potential of multiple comorbidities, the management of elderly patients presenting with gallstone ileus is usually challenging. The post-operative phase is also a crucial aspect of an elderly patient’s care, with increased rates of significant complications that include pneumonia and cardiac compromise [8]. Mortality rates for gallstone ileus used to be extremely high - reaching rates of more than 60% prior to 1925. More recently mortality rates have improved, ranging between 7% and 30% with an average mortality rate of 18% [7]. This high mortality rate was not only attributed to advanced age and associated comorbidities, but also to delayed presentation [8].

The term gallstone “ileus” itself is a misnomer. An ileus is defined as paralysis of the bowel’s peristaltic mechanism that eventually leads to a delay in the passage of enteric contents. The terminology of gallstone ileus refers to a mechanical obstruction caused by a lodged gallstone that has a sufficient diameter to cause an up-stream blockage. This is thought to be caused by impaction of a gallstone in the small bowel after passing through a biliary-enteric fistula [9]. This fistula may induce pneumobilia (in 34% of cases), bowel obstruction (70%) and an aberrantly located gallstone (35%) [10]. This was recognised radiologically and is referred eponymously as Rigler’s triad [11]. Approximately 75% of these fistulae are cholecystoduodenal, whereas only 10% to 20% are cholecystocolonic [12]. Approximately 40% to 50% of patients eventually diagnosed with gallstone ileus have a history of recent bouts of biliary colic, jaundice or acute cholecystitis [9].

The terminal ileum and the ileocaecal valve are narrow and have inefficient peristalsis. As a result, spontaneous gallstone evacuation in individuals with mechanical small bowel obstruction is uncommon, in particular when the gallstone is more than 2.5 cm in diameter [10].

There are two other rare subtypes of the already rare diagnosis of gallstone ileus: gallstone coleus and Bouveret’s syndrome [2]. Gallstone coleus is large bowel obstruction caused by a gallstone following its transit through a fistula to the bowel. It is an extremely rare cause of large bowel obstruction. Bouveret’s syndrome is an even rarer type of gallstone-associated gastrointestinal obstruction. This occurs when a gallstone lodges in the duodenum that subsequently leads to gastric outlet obstruction [2].

Traditional management of gallstone ileus involves a longitudinal enterotomy proximal to the obstructing calculus, removal of the offending stone and closure of the bowel (enterolithotomy) [13]. Following this initial management, consideration of a deferred cholecystectomy and fistula repair is deliberated. A one-stage procedure (i.e., enterolithotomy and concurrent repair of fistula) has been associated with higher complication rates when compared to performing the two procedures consecutively, with high morbidity (61% vs 27%) and mortality rates (17% vs 12%) [9]. Recently, laparoscopic and laparoscopic-assisted enterolithotomy have been used for the treatment of gallstone ileus and found to be more beneficial over open surgery in selected patients [1]. Indeed, the patient described in our case review was consented for a laparoscopic enterolithotomy +/- conversion to open procedure.

## Conclusions

Gallstone ileus is an uncommon cause of bowel obstruction and carries a high mortality due to the likelihood of associated advanced age, possible multiple comorbidities and late presentation. Management is usually surgical and the choice of surgical approach depends on preoperative assessment, associated comorbidities, intraoperative findings and the skill mix of the operating surgeon. Although less common, we present a case of spontaneous resolution of gallstone ileus. This highlights the crucial significance of the surgeon’s awareness of the possibility of uncommon presentations and outcomes, as well as the importance of adapting plans based on evolving clinical scenarios.
